# Complete Genome Sequences of 13 Bacillus subtilis Soil Isolates for Studying Secondary Metabolite Diversity

**DOI:** 10.1128/MRA.01406-19

**Published:** 2020-01-09

**Authors:** Heiko T. Kiesewalter, Carlos N. Lozano-Andrade, Gergely Maróti, Dan Snyder, Vaughn S. Cooper, Tue Sparholt Jørgensen, Tilmann Weber, Ákos T. Kovács

**Affiliations:** a Bacterial Interactions and Evolution Group, DTU Bioengineering, Technical University of Denmark, Kongens Lyngby, Denmark; b Institute of Plant Biology, Biological Research Center of the Hungarian Academy of Sciences, Szeged, Hungary; c Microbial Genome Sequencing Center, Pittsburgh, Pennsylvania, USA; d Department of Microbiology and Molecular Genetics, University of Pittsburgh, Pittsburgh, Pennsylvania, USA; e The Novo Nordisk Foundation Center for Biosustainability, Technical University of Denmark, Kongens Lyngby, Denmark; Indiana University, Bloomington

## Abstract

Bacillus subtilis is a plant-benefiting soil-dwelling Gram-positive bacterium with secondary metabolite production potential. Here, we report the complete genome sequences of 13 B. subtilis strains isolated from different soil samples in Germany and Denmark.

## ANNOUNCEMENT

Various species of the *Bacillus* genus have been exploited for biocontrol of crop plants. Bacillus subtilis is the most studied bacterium of the bacilli due to its high potential for industrial production of proteins, its utilization as a plant biological, and its easy genetic modification (1). In addition, the biofilm development of B. subtilis has been intensely investigated under laboratory settings (2–5) and during colonization of plant root (6–8) and fungal mycelia (9). The biocontrol potential of B. subtilis is determined by its ability to produce a variety of secondary metabolites, including surfactin, plipastatin (or fengycin), and bacillaene (10). Here, we performed complete genome sequencing of 13 B. subtilis strains in order to facilitate a detailed investigation of genes involved in secondary metabolite production.

B. subtilis strains were isolated from various sampling sites in Germany and Denmark (see details under BioProject accession number PRJNA587401) by using spore selection and specifically isolating architecturally complex colonies reminiscent of colony biofilm formation of B. subtilis (1, 5, 8, 11). Strains 73 and 75 were isolated specifically by inserting a constitutively produced green fluorescent protein (GFP) into the *amyE* gene, as described earlier (11). After biochemical assays, biofilm tests, and chemical analyses of natural products of the isolated strains, 13 B. subtilis strains were scrutinized with genome sequencing.

For Illumina sequencing, genomic DNA of the B. subtilis strains was isolated with the GeneMatrix bacterial and yeast genomic DNA purification kit according to the manufacturer’s instructions (EURx, Gdansk, Poland). Paired-end libraries were prepared for the strains, except MB9_B4, using the NEBNext Ultra II DNA library prep kit for Illumina (catalog number E7645L). Paired-end reads were generated on an Illumina NextSeq sequencer using a TG NextSeq 500/550 high-output kit v. 2 (300 cycles). In the case of MB9_B4, a mate pair library was generated using an Illumina Nextera mate pair kit (catalog number FC-132-1001) with insert sizes ranging from 6 to 15 kb. DNA sequencing was carried out on an Illumina MiSeq machine using V2 sequencing chemistry, resulting in 2 × 250-bp reads.

For Nanopore sequencing, genomic DNA was extracted using Qiagen blood and tissue kits (catalog number 69506), following the manufacturer’s protocol, using lysozyme digestion prior to extraction. This extra treatment was performed by resuspending the cell pellet in 200 μl of 20 mg/ml lysozyme and incubating the samples for 20 minutes at 37°C. Before sequencing on the Nanopore instrument, a ligation sequencing kit (catalog number SQK-LSK109) was used with native barcoding expansion 1-12 (catalog number EXP-NBD104) following the manufacturer’s protocol. Libraries were sequenced using an R9.4.1 flow cell and a MinION device running a 48-h sequencing cycle without base calling. The reads were base called and demultiplexed using Guppy v. 3.1.5 on an Amazon Web Service (AWS) GPU instance with quality control, as described before (12).

For *de novo* assembly, Illumina reads were adapter and quality trimmed using AdapterRemoval v. 2.1.7 (13) with the switches –trimns and –trimqualities. Nanopore reads were adapter and quality trimmed using Porechop v. 0.2.4 (14) and assembled with the Flye assembler v. 2.6 (15) with the switches –g 5m and –plasmids as suggested in a recent benchmark (16). Then, the Flye assembly graph, the trimmed Nanopore reads, and the trimmed Illumina reads from each sample were used as input for Unicycler v. 0.4.8-beta assembly with the switches –existing_long_read_assembly and –no_correct. Unicycler builds on several existing tools based around SPAdes assembly v. 3.13.0 (17), Pilon v. 1.22 (18), and SAMtools v. 1.9 (19). Assemblies were evaluated using the graph visualization software Bandage v. 0.8.1 (20) and BUSCO v. 3 (21) with the *Bacillales* ODB9 database to evaluate the core gene content of each genome.

The assembly produced 13 circularized chromosomes comprising 4,063,468 to 4,263,919 bases with a G+C content of 43.4 to 43.9%. Three isolates contained circular plasmids, each 84 kb in size. Automated annotation was performed using the NCBI Prokaryotic Genome Annotation Pipeline (Table 1).

**TABLE 1:** Accession numbers, assembly metrics, and annotated features of the sequenced B. subtilis strains isolated

Strain or plasmid	GenBank accession no.	Avg coverage (×) for:		Genome assembly size (bp)	G+C content (%)	No. of rRNAs	No. of tRNAs	No. of CDS^*a*^	Topology
		Illumina reads	Nanopore reads						
73	CP045826	595	263	4,166,516	43.7	30	86	4,203	Circular
75	CP045825	651	289	4,156,459	43.9	30	86	4,169	Circular
MB8_B1	CP045823	691	420	4,221,278	43.5	30	86	4,275	Circular
MB8_B7	CP045821	667	320	4,191,568	43.4	30	88	4,362	Circular
Plasmid pBs001	CP045822	519	393	84,033					Circular
MB8_B10	CP045824	602	550	4,225,362	43.5	30	86	4,289	Circular
MB9_B1	CP045820	636	517	4,263,919	43.5	30	86	4,320	Circular
MB9_B4	CP045819	138	480	4,105,407	43.8	30	86	4,108	Circular
MB9_B6	CP045818	671	440	4,087,720	43.8	30	86	4,089	Circular
P5_B1	CP045817	512	395	4,083,248	43.8	30	88	4,053	Circular
P5_B2	CP045816	586	353	4,103,324	43.6	30	86	4,245	Circular
P8_B1	CP045922	585	358	4,215,512	43.4	30	88	4,386	Circular
Plasmid pBs003	CP045923	510	260	84,215					Circular
P8_B3	CP045812	498	319	4,215,511	43.4	30	88	4,386	Circular
Plasmid pBs005	CP045813	386	490	84,215					Circular
P9_B1	CP045811	658	430	4,063,468	43.8	30	86	4,075	Circular

a CDS, coding sequences.

Genes coding for proteins possibly involved in secondary metabolite production were identified using antiSMASH v. 5 (22), which revealed the presence of gene clusters encoding surfactin (*srf*), plipastatin (*pps*), bacillaene (*pks*), and bacillibactin (*dhb*) biosynthesis in all isolates except strain P5_B2, which lacks the majority of the bacillaene (*pks*) biosynthetic gene cluster. Future detailed analysis of these biosynthetic gene clusters will be performed to reveal differences in secondary metabolite profiles.

### Data availability.

The raw data and assemblies have been deposited in GenBank under the BioProject accession number PRJNA587401. The complete genome sequence accession numbers are listed in Table 1.
